# Editorial: Oral mucosa and disorders: from drug discovery to development and translation

**DOI:** 10.3389/fphar.2024.1445126

**Published:** 2024-07-10

**Authors:** Vivek Ghate, Mohammed S. Alqahtani, Harsh Shah, K. M. Veena

**Affiliations:** ^1^ Yenepoya Technology Incubator, Yenepoya (Deemed to be University), Mangalore, Karnataka, India; ^2^ Department of Pharmaceutics, College of Pharmacy, King Saud University, Riyadh, Saudi Arabia; ^3^ J-STAR Research, Inc., South Plainfield, NJ, United States; ^4^ Department of Oral Medicine and Radiology, Yenepoya Dental College, Yenepoya (Deemed to be University), Mangalore, Karnataka, India

**Keywords:** oral cavity disorders, oral submucous fibrosis, oral lichen planus (OLP), oral cancer (OC), drug discovery and development, drug delivery and targeting, oral disease and systemic disease

## Introduction

Oral mucosal disorders represent a significant health challenge due to their prevalence across all demographics and the considerable impact they have on the quality of life. These disorders, which include conditions such as pemphigus, oral submucous fibrosis (OSF), and oral lichen planus (OLP), often result in chronic pain and difficulty in performing essential functions like eating, swallowing, and talking. The current mainstay of treatment involves the use of systemic or topical steroids, which, while effective, pose severe side effects and toxicities with prolonged use. The urgent need for alternative treatment options is evident, and recent research highlights promising directions for future therapeutic developments.

## Pemphigus and the potential of Chinese herbal medicine

Pemphigus is a life-threatening autoimmune disorder characterized by painful blistering of the skin and mucous membranes. Traditional Chinese herbal medicine (CHM) has emerged as a potential adjunctive therapy for managing this condition. A study conducted by Wu et al. involved a retrospective analysis of 1,037 pemphigus patients in Taiwan, comparing the outcomes of those who used CHM with those who did not. The findings were promising: CHM users exhibited a significantly lower overall mortality risk compared to non-users (HR: 0.422, 95% CI: 0.242–0.735, *p* = 0.0023). Moreover, the cumulative incidence of overall survival was significantly higher among CHM users (*p* = 0.0025, log rank test).

The study identified two main clusters of CHMs: one including Qi–Ju–Di–Huang–Wan, Dan–Shen, Jia–Wei–Xiao–Yao–San, Huang–Lian, and Di–Gu–Pi; and another comprising Jin–Yin–Hua and Lian–Qiao. These herbs, known for their anti-inflammatory and immunomodulatory properties, may offer a complementary approach to conventional steroid therapy. The reduction in mortality rates observed suggests that CHM could play a crucial role in the long-term management of pemphigus, warranting further clinical trials to explore its efficacy and safety comprehensively.

## Areca nut and oral submucous fibrosis: unraveling the mechanisms

Oral submucous fibrosis (OSF) is a pre-cancerous condition predominantly caused by the chewing of areca nut (betel quid). Arecoline, the principal alkaloid in areca nut, is known to induce fibroblast proliferation, but the specific molecular mechanisms have remained unclear. Research by Zhang et al. sheds light on this by demonstrating that arecoline enhances Transforming growth factor-β (TGF-β)-induced activation and fibrotic changes in buccal mucosal fibroblasts (BMFs).

## Oral cancer: the imperative of early detection and prevention

Oral cancer remains a critical health issue, with significant morbidity and mortality. According to Kumari et al., the incidence of oral cancer is rising, particularly in Asian countries, with factors such as genetic mutations, viral infections (notably HPV), and lifestyle habits (e.g., tobacco use, alcohol consumption) contributing to its occurrence and progression.

Early detection and management of oral potentially malignant disorders (OPMDs) are crucial in preventing their progression to cancer. OPMDs, including leukoplakia, erythroplakia, and OSF, are characterized by dysplastic changes that signify a high risk of malignancy. Advances in diagnostic techniques, such as molecular markers and imaging technologies, have improved the ability to detect these lesions early and assess their malignant potential. Enhanced screening programs and public awareness campaigns are essential to reduce the burden of oral cancer, emphasizing the need for ongoing research and development in this field.

## Antioxidants in oral lichen planus management

Oral lichen planus (OLP) is a chronic inflammatory condition of the oral mucosa, often associated with significant pain and discomfort. Traditional treatments involve corticosteroids, but their long-term use can lead to adverse effects. A meta-analysis by Bao et al. reviewed the efficacy and safety of antioxidant therapy in OLP management. The analysis included 17 studies with 704 patients, showing that antioxidants significantly reduced pain and clinical scores in OLP patients. Additionally, the pain and clinical resolution rates improved with antioxidant therapy, highlighting its potential as a safer alternative to steroids.

Antioxidants, by mitigating oxidative stress and inflammation, can offer symptomatic relief and improve the quality of life for OLP patients. This therapeutic approach shows the importance of exploring non-steroidal options for managing chronic oral mucosal disorders, aligning with the broader goal of minimizing treatment-related toxicities.

## Summary and outlook

The management of oral mucosal disorders remains a significant clinical challenge, exacerbated by the side effects and toxicities associated with long-term steroid use. The promising findings from recent studies on CHM, arecoline’s molecular mechanisms, early detection of OPMDs, and antioxidant therapy provide a hopeful outlook for developing safer and more effective treatments. Continued research is essential to translate these insights into clinical practice, ultimately improving the quality of life for individuals affected by these debilitating conditions. Collaborative efforts between researchers, clinicians, and policymakers are crucial to advancing therapeutic options and addressing the unmet needs in the treatment of oral mucosal disorders.

